# Intraocular inflammation secondary to intravitreal brolucizumab treated successfully with Sub-Tenon triamcinolone: A case report

**DOI:** 10.1016/j.ajoc.2022.101289

**Published:** 2022-01-22

**Authors:** Shree K. Kurup, Tarek Tabbaa, Jose J. Echegaray, Armando L. Oliver

**Affiliations:** a Case Western Reserve University-University Hospitals, Cleveland, OH, USA; b University of Puerto Rico, Medical Sciences Campus, School of Medicine, Department of Ophthalmology, San Juan, PR, USA

**Keywords:** Brolucizumab, Intraocular inflammation, Age-related macular degeneration, Sub-Tenon triamcinolone, Uveitis

## Abstract

**Purpose:**

To report on a case of successful treatment of intraocular inflammation (IOI) secondary to brolucizumab intravitreal injection that responded to a single sub-Tenon injection of triamcinolone.

**Observations:**

An 81-year-old female with a longstanding history of exudative age-related macular degeneration (AMD) was unresponsive to various regimens of *anti*-VEGF injections. Her AMD was treated with one intravitreal injection of brolucizumab (6mg/0.05ml) into the right eye. On a follow-up visit, she had a new-onset conjunctival injection, with anterior chamber and vitreous inflammation, in the right eye, which was diagnosed as non-granulomatous iridocyclitis. The patient was treated with one posterior sub-Tenon injection of triamcinolone (40mg/ml) into the right eye. Subsequently, there was a durable resolution of inflammation, and her vision improved along with the resolution of her exudation.

**Conclusions and Importance:**

This case suggests that some brolucizumab-related IOI episodes may be treated with posterior sub-Tenon triamcinolone. Further studies may serve to elucidate the role of sub-Tenon triamcinolone in brolucizumab-associated IOI.

## 1 Introduction

Brolucizumab is a novel anti-vascular endothelial growth factor (VEGF) therapy that has recently been approved in the United States to treat exudative age-related macular degeneration (AMD).^1^^,^^2^ It consists of a single-chain antibody fragment with a relatively small molecular weight, allowing it to be delivered at higher concentrations than the previously available treatment alternatives.^1^ Its advantages include a higher rate of exudation resolution and the potential to extend treatment intervals further than what is possible with the other available treatment alternatives.^1^^,^^2^

Recent reports of intraocular inflammation (IOI) after intravitreal injection of brolucizumab have raised concerns regarding its safety.^2^, ^3^, ^4^, ^5^, ^6^, ^7^ These IOI episodes vary in severity and may include perivascular hemorrhages, optic disk swelling, arterial sheathing, venous phlebitis, and occlusive vasculitis.^4^ An extended 96-week safety outcomes report from two phase III clinical trials revealed a 4.4% rate of intraocular inflammation, and post-marketing reports estimate the incidence of occlusive vasculitis to be 1–3 per 10,000 vials.^4^

Posterior sub-Tenon triamcinolone injections (PST) have been shown to be effective for the management of active intraocular inflammation.^8^ These injections are well-tolerated by patients, with only a minority of them developing complications such as glaucoma or cataracts.^8^^,^^9^ We hereby present a case of a woman with IOI following an intravitreal brolucizumab injection that was successfully treated with PST.

## 2 Case report

**Fig. 1:**
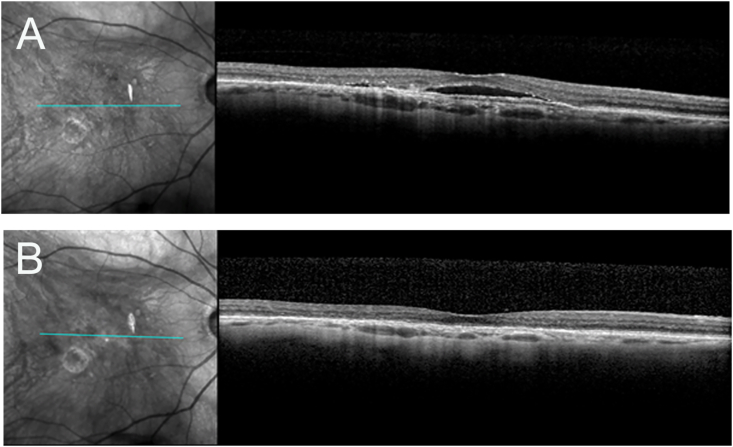
Spectral-domain optical coherence tomography of the right macula. A. Prior to treatment with brolucizumab, the images show evidence of chronically persistent subretinal fluid. B. Showing the complete resolution of subretinal fluid after treatment with brolucizumab and a subsequent posterior sub-Tenon triamcinolone injection.

An 81-year-old Caucasian female with an extensive history of exudative AMD had been treated with repeated intravitreal injections of various *anti*-VEGF agents with little success for six years. Her visual acuity over her disease course ranged from 20/40-20/60. The exudation varied but proved persistent and refractory to conventional therapy. The patient had no history of autoimmune or inflammatory conditions. During one of her follow-up visits, the patient's vision in the right eye at this visit was recorded as 20/50^−2^, and she was pseudophakic with no other ocular pathology. A macular ocular coherence tomography (OCT) revealed chronic subretinal fluid (Fig. 1A). The decision was made to begin treatment with brolucizumab (Beovu-Novartis 6 mg/.05 mL) to implement a new approach for the treatment of her condition. The patient was positioned and prepped in a sterile manner, and the injection was performed uneventfully in the right eye.

**Fig. 2:**
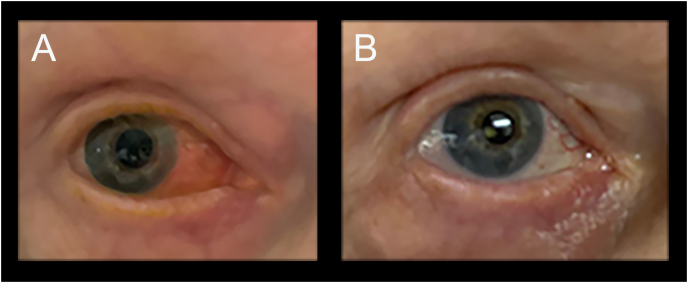
External color photographs of the right eye. A. Ten days following the intravitreal injection of brolucizumab, revealing marked conjunctival injection. B. Ten days following a posterior sub-Tenon injection of triamcinolone, showing the resolution of conjunctival injection. (For interpretation of the references to color in this figure legend, the reader is referred to the Web version of this article.)

**Fig. 3:**
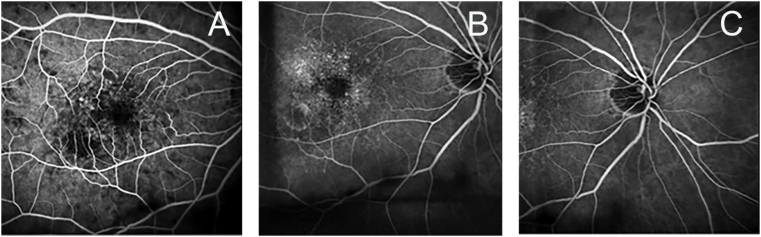
Fluorescein angiogram of the right posterior pole. The early (A) and late (B and C) arteriovenous phase images reveal the presence of an occult choroidal neovascular membrane and transit defects consistent with the diagnosis of exudative age-related macular degeneration. The angiography did not reveal any evidence of perivascular vascular leakage or ischemia, which findings would have suggested retinal vasculitis.

The patient presented ten days after the administration of brolucizumab with severe pain in her right eye. The best-corrected vision was recorded to be 20/80. On a slit-lamp examination, there was 3+ conjunctival injection, a 1+ cell and flare grade in the anterior chamber, and 0.5+ cell grade in the anterior vitreous (Fig. 2A). Of note, there was no corneal endothelitis, vitreous haze, vascular sheathing, or optic disc edema. The contralateral eye was unchanged. Fluorescein angiography done at this visit showed no evidence of retinal vasculitis (Fig. 3).

A decision was made to treat with PST to temporize the patient's inflammation. The patient was prepped and draped in a sterile manner, and 40 mg/mL of triamcinolone was injected into the posterior sub-Tenon space in the right eye. The patient followed up ten days later, and subjectively her pain had subsided, and the vision was noted to have improved to 20/25. On slit-lamp examination, there was no conjunctival injection, and the anterior chamber was noted to have no cell or flare (Fig. 2B). An OCT of the macula was done at this visit, which showed the resolution of the subretinal fluid (Fig. 1B). Three months following treatment with intravitreal brolucizumab, the patient developed recurrent subretinal fluid in the treated eye. This recurrence was treated with two monthly aflibercept injections; however, the subretinal fluid persisted. Subsequently, the patient received a second brolucizumab injection which was provided concurrently with 30 mg of oral prednisone as prophylaxis for inflammation. At her last examination, four months following the second brolucizumab injection, the patient remained without any evidence of recurrent subretinal fluid or IOI. Her intraocular pressures remained stable and within normal limits throughout her course of therapy.

## 3 Discussion

Brolucizumab is a relatively new medication for exudative AMD treatment that has been added to the ophthalmologist's arsenal of *anti*-VEGF regimens.^1^ The structure of the medication has been postulated to contribute to its efficacy in the treatment of this chronic and insidious disease.^10^ It is composed of a small molecule weighing 26 kDa that is not dependent on a bulky framework for stability.^10^ Older iterations of *anti*-VEGF medications weigh anywhere from 115 kDa (aflibercept) to up to 147 kDa (bevacizumab).^10^ This feature, amongst many other qualities, has been hypothesized to contribute to the increased efficacy of this medication compared to older *anti*-VEGF regimens.^10^ Our case highlights the potential advantages of brolucizumab treatment. It resolved the patient's recalcitrant subretinal fluid which had been recorded on OCT over the past 6 years. Furthermore, it also helped regain some of the patient's vision, which had been lost over the course of her disease process.

The etiology of the inflammation related to the medication is still a matter of debate and has been yet to be completely elucidated.^5^ Studies of serological samples of patients that have had severe reactions to brolucizumab, such as retinal vasculitis, have shown the existence of antibodies to the medication.^4^ This may cater to a more immunological basis for some patients' inflammatory responses to the medication. In our case, we hypothesize that the source of the patient's reaction to the injection was likely secondary to a type III or type IV hypersensitivity reaction rather than an allergic type of reaction.

The use of corticosteroids to treat IOI secondary to injection of brolucizumab has been recently proposed by an international panel of experts following a concise review of the existing medical literature.^2^ Their report also highlights the importance of using ultra-wide-field color photographs and angiography, as well as OCT and OCT angiography, to carefully evaluate patients with brolucizumab-associated IOI for the presence of retinal vasculitis and retinal vascular occlusive events.^2^^,^^7^ According to the expert panel, initial treatment considerations should include the intensive use of potent topical corticosteroids while having a low threshold to starting therapy with intravitreal or systemic corticosteroids.

The case presented shows a mild instance of IOI that was effectively treated with PST. This technique not only decreases the medication burden on the patient but also allows the surgeon to titrate the amount of steroid injected effectively and monitor patients accordingly. Additionally, it ensures that the entire course of medication is delivered and does not rely on patient adherence to a drop regimen.

A recent report highlighted a case of severe retinal vasculitis secondary to brolucizumab injection.^4^ The use of steroid eye drops as well as intravitreal corticosteroids were not shown to halt the progression of severe vasculitis in that case.^4^ However, it is possible that milder cases of IOI, such as ours, could be easily managed with corticosteroids. Whereas more severe forms of inflammation may need to be managed more aggressively, and clinicians may need to pursue alternative forms of treatment such as vitrectomy.^2^^,^^4^

Based on the expert panel's recommendations and a review of the existing medical literature, a renowned group of authors designed the eight-step “A BRAVE SAVE” protocol, which acronym stands for “avoiding brolucizumab-related adverse events by scrutinizing available evidence.^7^” The first step involves proper patient selection, avoiding brolucizumab in individuals with only one good seeing eye or a history of systemic autoimmune diseases.^7^ The second step is to consent patients regarding the potential risks of brolucizumab therapy, including the significant risk (1/200) of retinal vasculitis.^7^ The third step is to perform a careful pre-injection examination and avoid using brolucizumab if any signs of acute or chronic ocular inflammation are found.^7^ The fourth step is to administer brolucizumab following the prevailing intravitreal injection protocols.^7^ The fifth step is to instruct patients to immediately report any symptoms suggestive of inflammation such as new floaters, pain, a decline in vision, and light sensitivity.^7^ Likewise, office staff should be aware that such symptoms may occur in a delayed and unpredictable manner, and if present, should prompt an urgent evaluation.^7^ The sixth step is to individualize treatment and consider less frequent injection protocols early on the course of therapy.

The seventh step of the protocol is to manage patients with brolucizumab-associated IOI, retinal vasculitis, or retinal vascular occlusions, as highlighted by Baumal et al.^2^^,^^7^ Patients with mild cases of IOI without vascular involvement should be started on aggressive therapy with potent topical corticosteroids, noting that in patients with moderate to severe IOI or retinal vasculitis or retinal vascular occlusions intravitreal steroid injections along with systemic corticosteroid therapy should be considered.^2^^,^^7^ The eighth step of the protocol advises waiting until any IOI has subsided prior to restarting *anti*-VEGF therapy.^7^ Optimally, those who develop IOI secondary to brolucizumab should be started on an alternate *anti*-VEGF agent.^7^ An injection holiday and close monitoring should be considered for those patients who have shown stability of their neovascular (AMD).^7^

## 4 Conclusion

This case highlights the efficacy and value of brolucizumab for treating exudative AMD while also recognizing that IOI is a side effect ophthalmologists should be aware of and monitor. It also acknowledges a novel approach to treating mild cases of inflammation associated with brolucizumab using PST. Ophthalmologists who wish to pursue treatment with brolucizumab for their patients should familiarize themselves with the “A BRAVE SAVE” protocol, which may help to minimize their patients’ risk of secondary IOI and retinal vasculitis and retinal occlusive events.^7^

## Patient consent

Informed consent was obtained prior to performing the procedure, including permission for publication of all photographs and images included herein. This report does not contain any personal information that could lead to the identification of the patient.

## Funding

No funding or grant support was used for this case report.

## Authorship

All authors attest that they meet the current ICMJE criteria for authorship.

## Declaration of competing interest

The following authors have no conflicts of interest: SK, TT, JE, AO.
